# Prediction of Gadolinium Polynitrides at High Pressures as High-Energy-Density Materials

**DOI:** 10.3390/molecules30030733

**Published:** 2025-02-06

**Authors:** Ye Yang, Jiamei Song, Haodi Zhang, Zhihui Li, Shuang Liu, Yuanyuan Wang, Xiaomin Su

**Affiliations:** 1 State Key Laboratory of High Pressure and Superhard Materials, College of Physics, Jilin University, Changchun 130012, China; yangye22@mails.jlu.edu.cn (Y.Y.); songjm23@mails.jlu.edu.cn (J.S.); zhanghd22@mails.jlu.edu.cn (H.Z.); zhihui@jlu.edu.cn (Z.L.); liu_shuang@jlu.edu.cn (S.L.); 2 Department of Respiratory Medicine, The Second Hospital of Jilin University, 218 Ziqiang Street, Changchun 130041, China

**Keywords:** polymeric nitrogen, high pressure, high energy density, first principles

## Abstract

Pressure-induced nitrogen-rich compounds hold significant application prospects in high-energy-density materials. Utilizing first-principles calculations and swarm-intelligence structure search methods, we have identified ten new types of Gd-N compounds with different configurations, such as one-dimensional N-chains composed of N_6_ rings or N_8_ rings, and two-dimensional N-layers constructed of N_14_ rings, N_18_ rings, or N_18_ + N_6_ rings. Moreover, the predicted Gd-N compounds exhibit different magnetic properties, and a magnetic phase diagram is constructed in the pressure range of 0 to 200 GPa. Remarkably, the volumetric energy density (11.58–17.79 kJ/cm^3^) of Gd polynitrides with high nitrogen content, including *P*-1(I)-GdN_6_, *P*-1(II)-GdN_6_, *R*-3-GdN_8_, *C*2*mm*-GdN_9_, and *P*1-GdN_10_, surpassed that of TNT (7.05 kJ/cm^3^), making them promising candidates for energetic materials. The discovery of diverse chain-like and layered structures in the GdN*_x_* compounds highlights the role of gadolinium in inducing the diversity and complexity of nitrogen arrangements.

## 1. Introduction

Polymeric nitrogen is an environmentally friendly material with high energy density. The significant energy difference between the N≡N triple bond (954 kJ/mol) and both the N=N double bond (418 kJ/mol) and the N-N single bond (160 kJ/mol) means that polymeric nitrogen can release a substantial amount of energy when decomposed into nitrogen. Its energy density is 2–3 times greater than that of traditional explosives such as TNT, making it a promising candidate for applications in energy storage, propellants, and explosives [1,2]. Theoretically, several novel polymeric nitrogen structures have been identified, including network structures (*cg*-N, *Pnnm*, and CW) [3,4,5], layered structures (A7, ZS, BP, LB, LP, HLP, and PP) [6,7,8,9,10,11], chain structures (*ch* and *Cmcm*) [6,12], and cage structures (N_10_) [13]. Experimentally, cubic gauge nitrogen (*cg*-N) was synthesized by Eremets et al. in 2004, using a laser-heated diamond anvil cell at 2000 K and 110 GPa [14]. To obtain another three types of polymeric nitrogen (LP, HLP, BP), similar high-temperature and -pressure conditions (P > 100 GPa, T > 2000 K) are required [15,16,17]. However, these extreme synthesis conditions limit the practical applications of these materials and result in low stability.

Recent studies have demonstrated that polymeric nitrogen can be synthesized at lower pressures by introducing additional elements, while also enhancing stability. Experimentally, metal nitrides such as LiN_5_, K_2_N_6_, Mg_2_N_4_, CsN_5_, and *tr*-BeN_4_ have been synthesized at pressures of 45, 45, 50, 60, and 85 GPa, respectively [18,19,20,21,22]. Notably, LiN_5_, Mg_2_N_4_, and *tr*-BeN_4_ can be quenched at ambient conditions. Compared to pure polymeric nitrogen, these nitrogen-rich compounds indeed possess significantly lower synthesis pressures, which is also conducive to the discovery of new polymeric nitrogen structures. Theoretically, polynitrides induced by main group elements and transition metals exhibit a variety of configurations, including azides [23,24], nitrogen rings (N_5_ and N_6_ rings) [25,26,27,28,29], nitrogen chains [20,30,31,32], and nitrogen layers [33,34,35,36]. Recently, lanthanide metals have garnered much attention due to the high localization of f-electrons, resulting in more diverse structures. Lanthanide polynitrides *P*6*mm*-CeN_14_ and *Imm*2-LaN_10_ feature intriguing structures such as two-dimensional (2D) molecular sieve structures and one-dimensional (1D) N_10_ cages, respectively [35,37]. Notably, *P*6*mm*-CeN_14_ possesses the highest energy density (8.45 kJ/g) among known metal polynitrides.

Gadolinium is a unique lanthanide metal with a complex electronic configuration (4f^7^5d^1^6s^2^), which stands out among lanthanide metals due to its theoretically largest magnetic moment of 7 μB/Gd^3+^. The combination of these two advantages enables Gd to induce new structures with unique configurations and magnetic properties, making it an outstanding candidate for coordination elements. Herein, we employ the first-principles evolutionary crystal structure search method to predict Gd-N compounds at pressures of 100 and 200 GPa. Five stable phases (*P*4/*nmm*-GdN, *P*-1(I)-GdN_3_, *P*-1(II)-GdN_3_, *P*2/*c*-GdN_5_, and *P*-1-GdN_5_) and five metastable phases (*P*-1(I)-GdN_6_, *P*-1(II)-GdN_6_, *R*-3-GdN_8_, *C*2*mm*-GdN_9_, and *P*1-GdN_10_) have been predicted. The volumetric energy density (11.58–17.79 kJ/cm^3^) of *P*-1(I)-GdN_6_, *P*-1(II)-GdN_6_, *R*-3-GdN_8_, *C*2*mm*-GdN_9_, and *P*1-GdN_10_ exceeds that of TNT (7.05 kJ/cm^3^), indicating that they are potential high-energy-density materials (HEDMs). The results enrich the magnetic phase diagram of the Gd-N system and lead to potential applications with energetic and magnetic properties.

## 2. Results and Discussion

### 2.1. Structure Prediction and Stability Analysis

**Figure 1:**
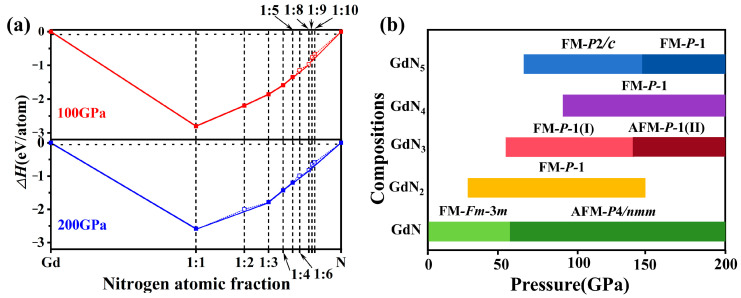
(**a**) The convex hulls of the Gd-N system constructed from the enthalpy difference relative to the Gd and nitrogen. The solid and blank squares represent the structures that fall on and fall out of the convex hulls, respectively. (**b**) Pressure–composition phase diagram of the Gd-N compounds within the range of 0–200 GPa.

A structure prediction for GdN*_x_* (*x* = 1, 2, 3, 4, 5, 6, 8, 9, and 10) compounds is performed to obtain novel Gd-N high-pressure phases at 100 and 200 GPa. As shown in Figure 1a,b, the convex hulls and phase diagram of the Gd-N system from 0 to 200 GPa are plotted according to the formation enthalpies, defined as ΔH = [H(GdN*_x_*) − H(Gd) − *x*H(N)]/(1 + *x*). The *C*2/*m* phase of Gd and the *Pa*-3-N_2_, *P*4_1_2_1_2-N, *P*2_1_/*c*-N, *R*-3*c*-N, *cg*-N, and *Pba*2-N of N are selected as the reference phases [11,38,39,40]. Thermodynamically stable phases are denoted by solid squares, and unstable phases are represented by hollow squares. Besides three stable phases (*Fm*-3*m*-GdN, *P*-1-GdN_2_, and *P*-1-GdN_4_) reported in a previous study [41], we predicted five new stable Gd polynitrides (*P*4/*nmm*-GdN, *P*-1(I)-GdN_3_, *P*-1(II)-GdN_3_, *P*2/*c*-GdN_5_, and *P*-1-GdN_5_) at high pressure. For the GdN stoichiometry, a new phase *P*4*/nmm*-GdN was predicted at 100 GPa. Compared with the reported *Fm*-3*m* phase, *P*4*/nmm*-GdN is energetically more favorable within the range of 55–200 GPa (Figure S1a). For the reported *P*-1-GdN_2_ and *P*-1-GdN_4_, the stable pressure ranges are 27–155 GPa and 90–200 GPa, respectively (Figure S1b,d). In the GdN_3_ stoichiometry, *P*-1(I)-GdN_3_ and *P*-1(II)-GdN_3_ are predicted at 100 and 200 GPa, respectively. The *P*-1(I) phase remains stable at 54–140 GPa, then transforms to a *P*-1(II) phase (Figure S1c). For the GdN_5_, *P*2*/c* and *P*-1 phases are predicted at 100 and 200 GPa, respectively. The *P*2/*c* phase is stable above 65 GPa and then changes to a *P*-1 phase at 135 GPa (Figure S1e). Moreover, the calculations of phonon spectra show that five new Gd-N compounds are dynamically stable at the predicted pressure owing to the absence of an imaginary frequency in the Brillouin zone (Figure S2a–e). The phonon density of states (PHDOS) shows that the high-frequency vibrational modes arise from the vibration of N atoms, while the low-frequency vibration modes are primarily attributed to the collective motion of both Gd and N atoms (Figures S2 and S3). In addition, *P*-1(I)-GdN_3_ and *P*2*/c*-GdN_5_ also possess dynamical stability at ambient pressure.

With an increase in nitrogen content, five metastable Gd polynitrides (*P*-1(I)-GdN_6_, *P*-1(II)-GdN_6_, *R*-3-GdN_8_, *C*2*mm*-GdN_9_, and *P*1-GdN_10_) are proposed at 100 GPa or 200 GPa. The calculated phonon spectra verify their dynamic stability under the predicted pressure (Figure S3a–e). To explore their synthesis conditions and phase transition pressure, the enthalpy differences are separately calculated. For the GdN_6_ stoichiometry, a previously reported *P*-1-GdN_6_ is energetically more favorable above 36 GPa compared with the GdN and nitrogen [41]. As the pressure increases, *P*-1(I)-GdN_6_ is more stable at 61–192 GPa, then transforms to *P*-1(II)-GdN_6_ (Figure S4a). *R*-3-GdN_8_, *C*2*mm*-GdN_9_, and *P*1-GdN_10_ are energetically more favorable relative to the mixture of GdN and nitrogen at 50, 77, and 72 GPa, respectively (Figure S4b), suggesting that they can be synthesized by the path GdN + (*x* − 1)/2N_2_ → GdN*_x_* at these pressures.

### 2.2. Crystal Structure

**Figure 2:**
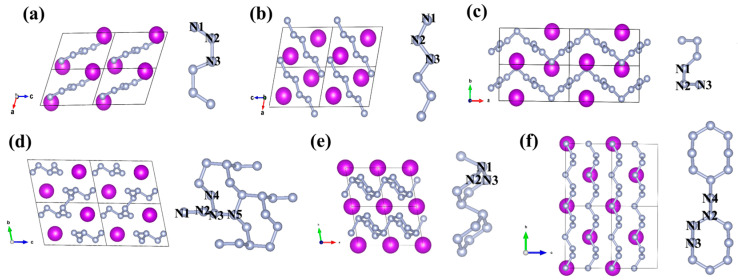
Crystalline structure and polymeric nitrogen unit of (**a**) *P*-1(I)-GdN_3_, (**b**) *P*-1(II)-GdN_3_, (**c**) *P*2*/c*-GdN_5_, (**d**) *P*-1-GdN_5_, (**e**) *P*-1(II)-GdN_6_, and (**f**) *C*2*mm*-GdN_9_. The purple and gray spheres represent Gd and N atoms, respectively.

**Figure 3:**
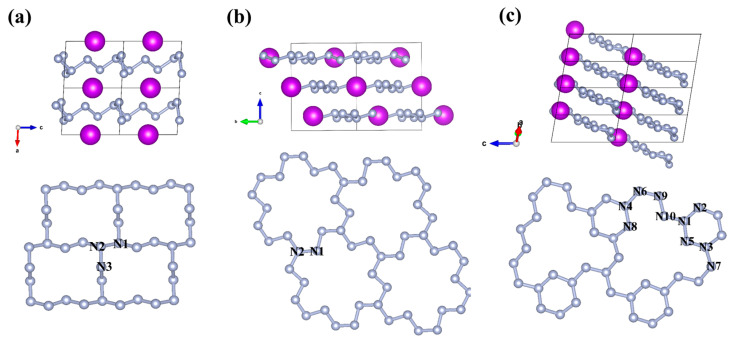
Crystalline structure and polymeric nitrogen unit of *P*-1(I)-GdN_6_ (**a**) at 100 GPa, *R*-3-GdN_8_ (**b**), and *P*1-GdN_10_ (**c**) at 200 GPa. The purple and gray spheres represent Gd and N atoms, respectively.

Various chain-like and layered structures have been predicted in GdN*_x_* compounds, with a wide range of configurations. For the chain-like structure, armchair and zigzag N_6_ chains are predicted in *P*-1(I)-GdN_3_ and *P*-1(II)-GdN_3_, respectively, while curved N_10_ chains are predicted in *P*2*/c*-GdN_5_ (Figure 2a–c). Beyond these nitrogen atomic chains, several novel nitrogen chains composed of nitrogen rings as building blocks have also been identified. For instance, in *P*-1-GdN_5_, eight nitrogen atoms are connected to construct a N_8_ ring, with two N_8_ rings sharing two nitrogen atoms to extend along the *a*-axis. Additionally, N_2_ units are connected on either side of the N_8_ rings (Figure 2d). In *P*-1(II)-GdN_6_, the chain structures consist of N_6_ rings, while the nitrogen atoms at both ends interlink extending along the *a*-axis (Figure 2e). Additionally, in *C*2*mm*-GdN_9_, two N_8_ rings are linked by a N atom in the middle to form the 1D infinite nitrogen chain along the *b*-axis (Figure 2f). For the layered structure, nitrogen atoms in *P*-1(I)-GdN_6_ form a layered structure composed of N_14_ rings, with each Gd atom located at the center of a N_14_ ring (Figure 3a), which is similar to *P*-1-CeN_6_ [42]. In *R*-3-GdN_8_, the polymeric nitrogen layer consists of folded N_18_ rings stacked in the ABC form along the *c*-axis, with Gd atoms at the center of a N_18_ ring (Figure 3b). This stacked arrangement of N_18_ rings has been previously reported in *R*-3-CeN_8_ [43]. Interestingly, the layered configuration consisting of both N_18_ and N_6_ rings was first found in *P*1-GdN_10_ (Figure 3c).

In contrast to the polymeric structures discussed above, *P*4*/nmm*-GdN contains non-bonded nitrogen atoms, and each Gd atom is coordinated with four N atoms (Figure S6). In *P*-1(I)-GdN_3_, *P*-1(II)-GdN_3_, *P*2*/c*-GdN_5_, *P*-1-GdN_5_, *P*-1(I)-GdN_6_, *P*-1(II)-GdN_6_, *R*-3-GdN_8_, *C*2*mm*-GdN_9_, and *P*1-GdN_10_, each Gd atom is coordinated by eight, ten, six, eight, eight, six, fourteen, ten, and six N atoms, respectively (Figure S7a–i). In addition, the N-N bond lengths in each structure are listed in Table S1. The lattice parameters of predicted GdN*_x_* compounds are provided in Tables S2 and S3.

### 2.3. Magnetic Properties

The magnetic states of GdN*_x_* compounds under different pressures have been systematically investigated. The energy relationships and ground states of the non-ferromagnetic (NM), ferromagnetic (FM), and anti-ferromagnetic (AFM) spin configurations are determined (Figure S8a–i). The results show that the energy of the NM configurations is on average 10 eV/f.u higher than that of FM and AFM spin configurations, indicating that the GdN*_x_* compounds are all magnetic. In the stable phases *Fm*-3*m*-GdN, *P*-1-GdN_2_, *P*-1(I)-GdN_3_, *P*-1-GdN_4_, *P*2/*c*-GdN_5_, and *P*-1-GdN_5_, the energy of the FM state is more favorable, which indicates an FM character. In addition, the metastable phases *P*-1(I)-GdN_6_, *P*-1(I)-GdN_6_, and *C*2*mm*-GdN_9_ also exhibit FM properties at 0–200 GPa. The total magnetic moment of each FM phase is 6.9 µB, and the local magnetic moment of each Gd atom is 6.6 µB at 0 GPa, suggesting that the magnetism mainly comes from Gd atoms.

**Figure 4:**
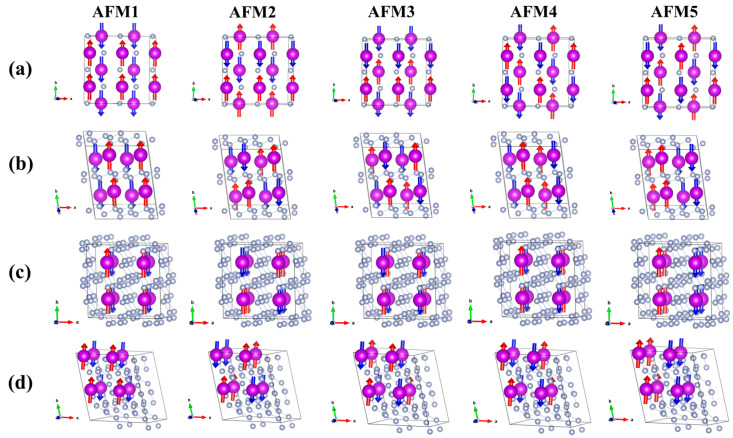
Different anti-ferromagnetic configurations of (**a**) *P*4*/nmm*-GdN, (**b**) *P*-1(II)-GdN_3_, (**c**) *R*-3-GdN_8_, and (**d**) *P*1-GdN_10_. The purple and gray spheres represent Gd and N atoms, respectively. The red arrow and blue arrow represent that the magnetism is along the (0, 1, 0) and (0, −1, 0) direction, respectively.

Additionally, *P*4/*nmm*-GdN, *P*-1(II)-GdN_3_, *R*-3-GdN_8_, and *P*1-GdN_10_ exhibit AFM properties. We consider five different AFM spin configurations through the antiparallel spin arrangement. For *P*4/*nmm*-GdN and *P*-1(II)-GdN_3_, AFM1 and AFM3 have the lowest ground-state energy, respectively (Figure 4a,b). For *R*-3-GdN_8_, the ground-state AFM structure is AFM1 and AFM5 at 23–200 GPa, as shown in Figure 4c. It is worth mentioning that the energy gap between AFM1 and AFM5 is less than 1 meV/f.u. Such a small energy difference indicates that the ground-state spin structure of *R*-3-GdN_8_ may be more complex than that of other structures. Parallelly, for the *P*1-GdN_10_, the most stable configuration is AFM3 at 40–200 GPa, and may consist of a mixture of AFM1 and AFM3 at 19–40 GPa (Figure 4d).

### 2.4. Electronic Structures

**Figure 5:**
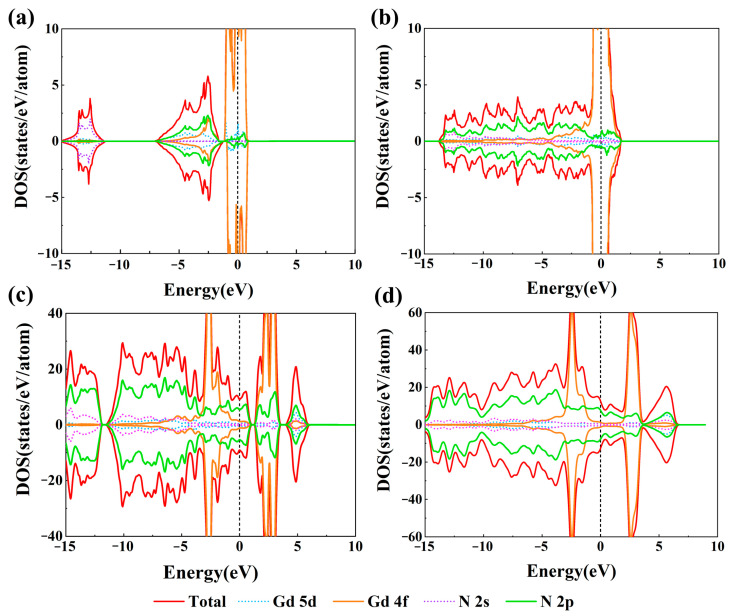
The total density of states (TDOS) and project density of states (PDOS) of (**a**) *P*4*/nmm*-GdN, (**b**) *P*-1(II)-GdN_3_, (**c**) *R*-3-GdN_8_, and (**d**) *P*1-GdN_10_.

**Figure 6:**
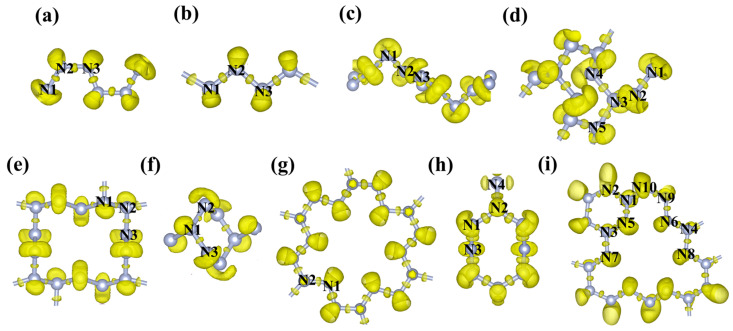
ELF of nitrogen structures of (**a**) *P*-1(I)-GdN_3_, (**b**) *P*-1(II)-GdN_3_, (**c**) *P*2/*c*-GdN_5_, (**d**) *P*-1-GdN_5_, (**e**) *P*-1(I)-GdN_6_, (**f**) *P*-1(II)-GdN_6_, (**g**) *R*-3-GdN_8_, (**h**) *C*2*mm*-GdN_9_, and (**i**) *P*1-GdN_10_ at predicted pressure.

To investigate the electronic properties of predicted GdN*_x_* compounds, we calculated their spin-resolved total density of states (TDOS) and projected density of states (PDOS). As shown in Figure 5a–d and Figure S9a–f, all the GdN*_x_* compounds are metallic phases. The DOS near the Fermi level is mainly contributed by the N_2p and Gd_4f orbital electrons. In addition, the PDOS for *P*4*/nmm*-GdN, *P*-1(II)-GdN_3_, *R*-3-GdN_8_, and *P*1-GdN_10_ exhibited symmetry between the spin-up and spin-down states, further confirming their AFM properties, as illustrated in Figure 5a–d. To further illustrate the bonding features and bonding strength of these compounds, the projection of the COHP (pCOHP) and integral of the COHP (ICOHP) are calculated. The pCOHP curve indicates that the bonding states of the N-N bond are fully occupied, and anti-bonding states are partially occupied. In Figures S10a–e and S11a–e, in the structures except for unbonded *P*4*/nmm*-GdN, the ICOHP values of N-N bonds are between 5.7 and 7.1, corresponding to the covalent bond interaction. The ICOHP values of Gd-N bonds are between 0.8 and 1.2, corresponding to the ionic bond interaction. The hybridization feature can be observed in the electron localization function (ELF) (Figure 6a–i). The strong localization of electrons between the N atoms represents the typical covalent N-N bond, in agreement with the COHP analysis. The nitrogen atoms in *P*-1(I)-GdN_3_, *P*-1(II)-GdN_3_, and *P*2*/c*-GdN_5_ are all sp^2^ hybridized, in which the hybrid orbital contains two σ bonds and a lone pair. In the *P*-1-GdN_5_, *P*-1(I)-GdN_6_, *P*-1(II)-GdN_6_, *R*-3-GdN_8_, *C*2*mm*-GdN_9_, and *P*1-GdN_10_, the 3-coordinated nitrogen atoms are in sp^3^ hybridization with three σ bonds and a lone pair, while 2-coordinated nitrogen atoms are hybrid in the sp^2^ state with two σ bonds and a lone pair.

### 2.5. Energy Density

The energy densities of GdN*_x_* compounds are calculated by decomposing into *Fm*-3*m*-GdN and *Pa*-3-N_2_. It can be seen from Table 1 that gravimetric energy density (*E_d_*) and volumetric energy densities (*E_v_*) gradually increase with an increase in nitrogen content. Five Gd polynitrides with high nitrogen content have a higher *E_v_* than TNT (7.05 kJ/cm^3^) [44], including *P*-1(I)-GdN_6_ (11.58 kJ/cm^3^), *P*-1(II)-GdN_6_ (7.94 kJ/cm^3^), *R*-3-GdN_8_ (11.95 kJ/cm^3^), *C*2*mm*-GdN_9_ (16.31 kJ/cm^3^), and *P*1-GdN_10_ (17.79 kJ/cm^3^). Moreover, *R*-3-GdN_8_, *C*2*mm*-GdN_9_, and *P*1-GdN_10_ possess high gravimetric energy density (2.77–3.98 kJ/g). Therefore, these Gd polynitrides are potential high-energy-density candidates with promising applications in industrial manufacturing, particularly in the fields of civilian fuels, explosives, and rocket propellants.

Several Gd-N compounds with the same stoichiometry are compared and analyzed. For the *P*-1(I)-GdN_3_ and *P*-1(II)-GdN_3_, which possess chain-like structures consisting of N atoms, their energy density (*E_d_* and *E_v_*) is almost the same. For the two GdN_5_ phases with chain-like structures, the energy density of the *P*2*/c* phase, which is composed of nitrogen rings, is higher than that of the *P*-1 phase, which is polymerized by nitrogen atoms. Similarly, the *E_d_* and *E_v_* of *P*-1(I)-GdN_6_ with a layered structure composed of N_14_ rings are larger than those of *P*-1(II)-GdN_6_ with chain-like N_6_ rings. Therefore, the configuration of nitrogen atoms plays a crucial role in determining the energy properties of polynitrides.

## 3. Conclusions

In summary, we systematically studied the various stoichiometric structures of the GdN*_x_* compounds within the pressure range of 0 to 200 GPa, utilizing the particle swarm optimization algorithm and density functional theory. Five new stable phases (*P*4*/nmm*-GdN, *P*-1(I)-GdN_3_, *P*-1(II)-GdN_3_, *P*2*/c*-GdN_5_, and *P*-1-GdN_5_) are discovered. Stability analysis indicates that *P*-1(I)-GdN_3_ and *P*2/c-GdN_5_ can be recovered at ambient pressure. In addition, five nitrogen-rich metastable phases (*P*-1(I)-GdN_6_, *P*-1(II)-GdN_6_, *R*-3-GdN_8_, *C*2*mm*-GdN_9_, and *P*1-GdN_10_) are identified, which can be synthesized at 61, 192, 50, 77, and 72 GPa, respectively. The analysis of magnetic properties reveals that *P*4*/nmm*-GdN, *P*-1(II)-GdN_3_, *R*-3-GdN_8_, and *P*1-GdN_10_ exhibit AFM properties at the pressure ranges of 55–200, 140–200, 23–200, and 19–200 GPa, respectively, while the other phases are FM phases. A high-pressure magnetic phase diagram of the Gd-N system is constructed. Additionally, the volume energy density of *P*-1(I)-GdN_6_ (11.58 kJ/cm^3^), *P*-1(II)-GdN_6_ (7.94 kJ/cm^3^), *R*-3-GdN_8_ (11.95 kJ/cm^3^), *C*2*mm*-GdN_9_ (16.31 kJ/cm^3^), and *P*1-GdN_10_ (17.79 kJ/cm^3^) is higher than that of TNT, making them potentially useful for applications in energetic materials and propellants. Overall, the presence of Gd induces a variety of configurations, providing a platform for exploring novel nitrogen-rich structures and understanding the relationship between energy density and configurations.

## 4. Computational Details

The structure searches were performed using CALYPSO code based on the particle swarm–intelligence structure prediction method [45,46,47]. The simulation cells of GdN*_x_* contain 1 and 2 formula units (f.u). The structural optimization and property calculations were implemented using the Vienna ab initio simulation package (VASP) and density functional theory with the projector-augmented wave pseudopotential method [48,49]. The exchange-correlation effects are described by the Perdew–Burke–Ernzerhof (PBE) function with generalized gradient approximation (GGA) [50]. The 4f^7^5d^1^6s^2^ and 2s^2^2p^3^ were treated as the valence electrons of the Gd and N atoms in the projected augmented wave (PAW) pseudopotentials. It is necessary for rare-earth metal nitrides to undergo local spin density approximation with Hubbard U corrections (GGA + U) to accurately describe the effective on-site electron–electron interaction of the f orbitals. A Hubbard U value of 6 eV was set in the calculations, supported by several theoretical studies demonstrating that DFT calculations with U = 6 eV show good agreement with experimental valence band X-ray photoelectron spectroscopy (XPS) results [51,52]. The plane-wave energy cutoff was set to 520 eV, and the Monkhorst–Pack *k* mesh spacing was set to 0.03 Å^−1^ [53]. The phonon calculations were performed by the PHONOPY program for all structures [54]. The electron localization function (ELF) was used to analyze the bonding environment and the degree of electron localization [55]. The crystal orbital Hamilton population (COHP) implemented in the LOBSTER package was used to quantitatively characterize the atomic interactions [56].

## Figures and Tables

**Table 1:** The mass density (*ρ*), gravimetric energy density (*E_d_*), and volumetric energy density (*E_v_*) of Gd-N compounds and TNT.

Structures	*ρ* (g/cm^3^)	*E_d_* (kJ/g)	*E_v_* (kJ/cm^3^)
*P*-1(I)-GdN_3_	7.29	0.36	2.62
*P*-1(II)-GdN_3_	7.15	0.35	2.52
*P*2/*c*-GdN_5_	6.10	1.15	9.53
*P*-1-GdN_5_	5.86	1.18	7.64
*P*-1(I)-GdN_6_	5.95	1.77	11.58
*P*-1(II)-GdN_6_	5.38	1.45	7.94
*R*-3-GdN_8_	5.07	2.21	11.95
*C*2*mm*-GdN_9_	4.84	3.33	16.31
*P*1-GdN_10_	4.76	3.41	17.79
TNT	1.64	4.30	7.05

## Data Availability

Dataset available on request from the authors.

## Supplementary Materials

The following supporting information can be downloaded at: https://www.mdpi.com/article/10.3390/molecules30030733/s1, Figure S1: enthalpy-pressure diagrams; Figure S2: The phonon dispersion spectra of (a) FM-P4/nmm-GdN, (b) FM-P-1(I)-GdN3, and (d) FM-P2/c-GdN5 at 100 GPa, (c) AFM-P-1(II)-GdN3 and (e) FM-P-1-GdN5 at 200 GPa; Figure S3: The phonon dispersion spectra of (a) P-1(I)-GdN6 at 100 GPa, (b) P-1(II)-GdN6, (c) R-3-GdN8, (d) C2mm-GdN9, and (e) P1-GdN10 at 200 GPa; Figure S4: Calculated formation enthalpy with respect to reference states GdN and nitrogen; Figure S5: The phonon spectra of (a) P-1(I)-GdN3 and (b) P2/c-GdN5 at 0 GPa; Figure S6: Crystalline structure and polyhedron unit of GdN; Figure S7: polyhedron unit. Figure S8. The enthalpy values of the ferromagnetic phase, antiferromagnetic and non-ferromagnetic phases of GdN (a), GdN2 (b), GdN3 (c), GdN4 (d), GdN5 (e), GdN6 (f), GdN8 (g), GdN9 (h), and GdN10 (i) at different pressures; Figure S9: The total density of states (TDOS) and project density of states (PDOS) of (a) P-1(I)-GdN3, (b) P2/c-GdN5, (c) P-1-GdN5, (d) P-1(I)-GdN6, (e) P-1(II)-GdN6, and (f) C2mm-GdN9 at predicted pressure; Figure S10: The -pCOHP and -ICOHP between Gd-N and N-N of Fm-3m-GdN (a), P-1(I)-GdN3 (b), P-1(II)-GdN3 (c), P2/c-GdN5 (d), and P-1-GdN5 (e) at predicted pressure; Figure S11: The -pCOHP and -ICOHP between Gd-N and N-N of P-1(I)-GdN6 (a) at 100 GPa, P-1(II)-GdN6 (b), R-3-GdN8 (c), C2mm-GdN9 (d), and P1-GdN10 (e) at predicted pressure; Table S1: The N-N bond lengths of Gd-N compounds; Tables S2: Structural parameters of predicted stable GdNx (n=1, 3, 5) compounds; Table S3: Structural parameters of predicted metastable GdNx (n=6, 8, 9, 10).
