# Supplementary material for: Prediction of Gadolinium Polynitrides at High Pressures as High-Energy-Density Materials
Source: Molecules. 2025 Feb 6;30(3):733. doi: 10.3390/molecules30030733 (PMC11819825; doi:10.3390/molecules30030733)
Supplement: Supplementary file 1 [file molecules-30-00733-s001.zip › molecules-3398354-supplementary.pdf]

Supplementary Material

Prediction of Gadolinium Polynitrides at High Pressures as High-Energy-Density Materials

Ye Yang [1], Jiamei Song [1], Haodi Zhang [1], Zhihui Li [1], Shuang Liu [1], Yuanyuan Wang [1,*], and Xiaomin Su [2,*]

[1] State Key Laboratory of High Pressure and Superhard Materials, College of Physics, Jilin University,
Changchun 130012, China; yangye22@mails.jlu.edu.cn (Y.Y.); songjm23@mails.jlu.edu.cn (J.S.); zhanghd22@mails.jlu.edu.cn (H.Z.); zhihui@jlu.edu.cn (Z.L.); liu_shuang@jlu.edu.cn (S.L.)

[2] Department of Respiratory Medicine, The Second Hospital of Jilin University, 218 Ziqiang Street,
Changchun 130041

* Correspondence: yy_wang@jlu.edu.cn (Y.W.); China; suxiaomin@jlu.edu.cn (X.S.)

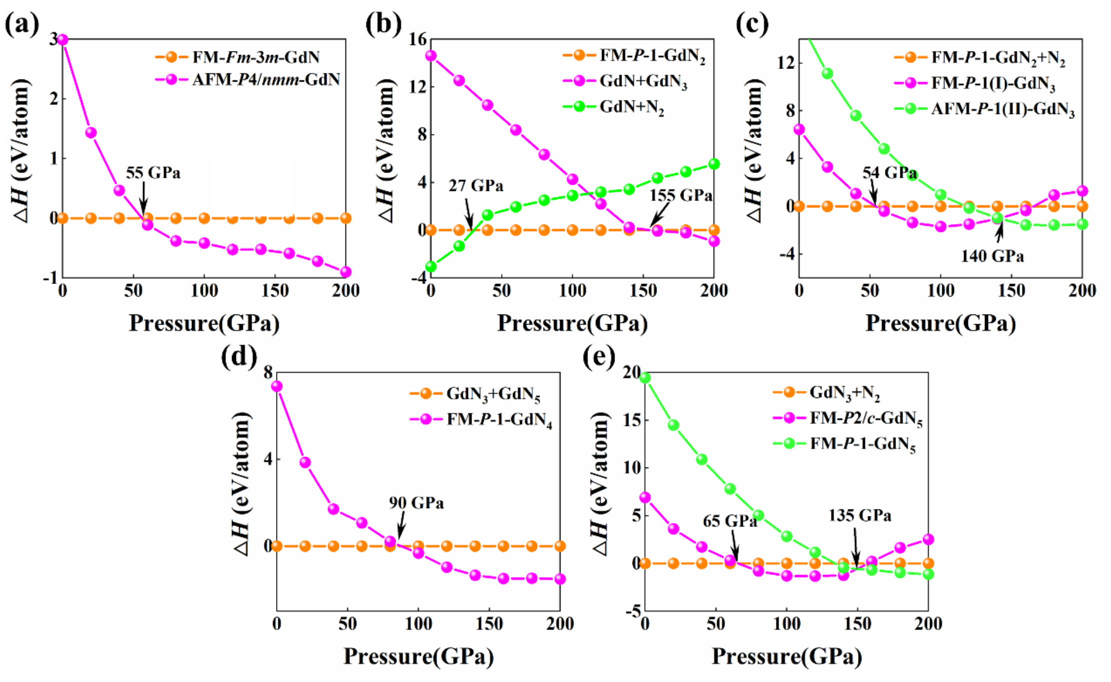

Figure S1. Enthalpy-pressure diagrams of (a) GdN, (b) GdN_2, (c) GdN_3, (d) GdN_4 and (e) GdN_5.

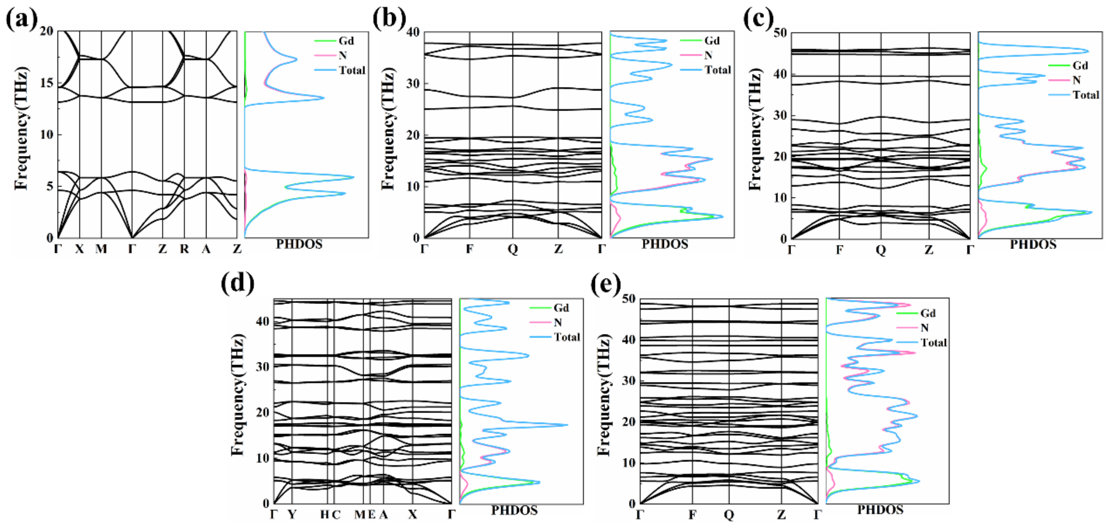

Figure S2. The phonon dispersion spectra of (a) FM-*P*4/*nmm*-GdN, (b) FM-*P*-1(I)-GdN_3, and (d) FM-*P*2/*c*-GdN_5 at 100 GPa, (c) AFM-*P*-1(II)-GdN_3 and (e) FM-*P*-1-GdN_5 at 200 GPa.

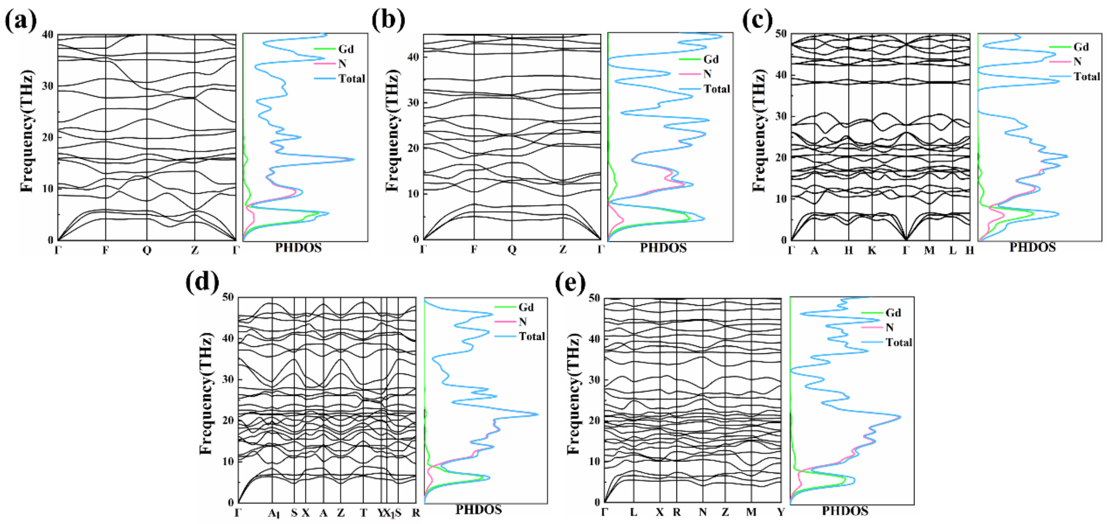

Figure S3. The phonon dispersion spectra of (a) *P*-1(I)-GdN_6 at 100 GPa, (b) *P*-1(II)-GdN_6, (c) *R*-3-GdN_8, (d) *C*2*mm*-GdN_9, and (e) *P*1-GdN_{10} at 200 GPa.

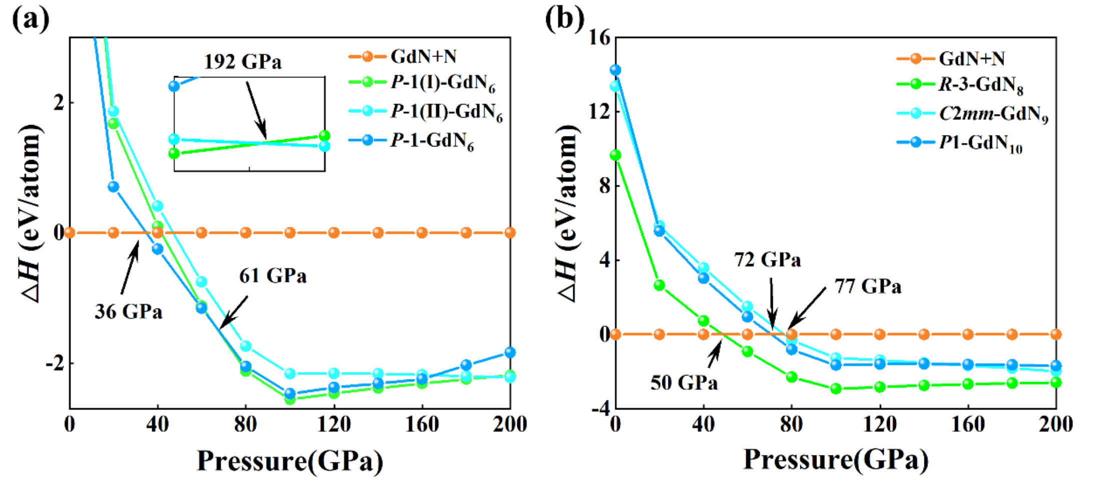

Figure S4. Calculated formation enthalpy with respect to reference states GdN and nitrogen.

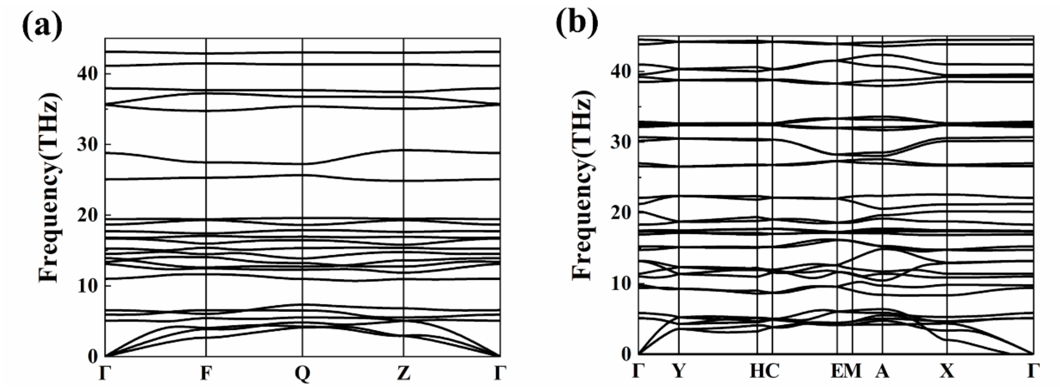

Figure S5. The phonon spectra of (a) *P*-1(I)-GdN_3 and (b) *P*2/*c*-GdN_5 at 0 GPa.

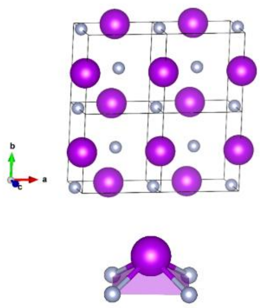

Figure S6. Crystalline structure and polyhedron unit of GdN.

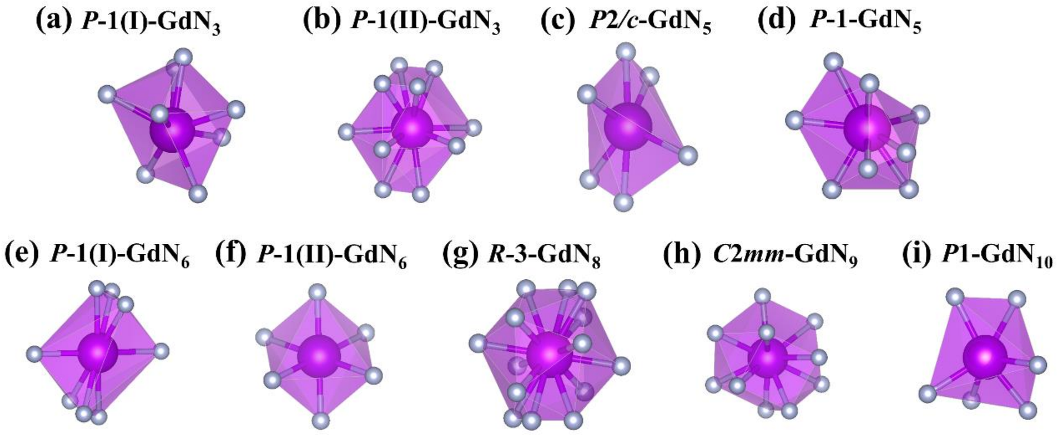

Figure S7. The polyhedron unit of Gd-N system.

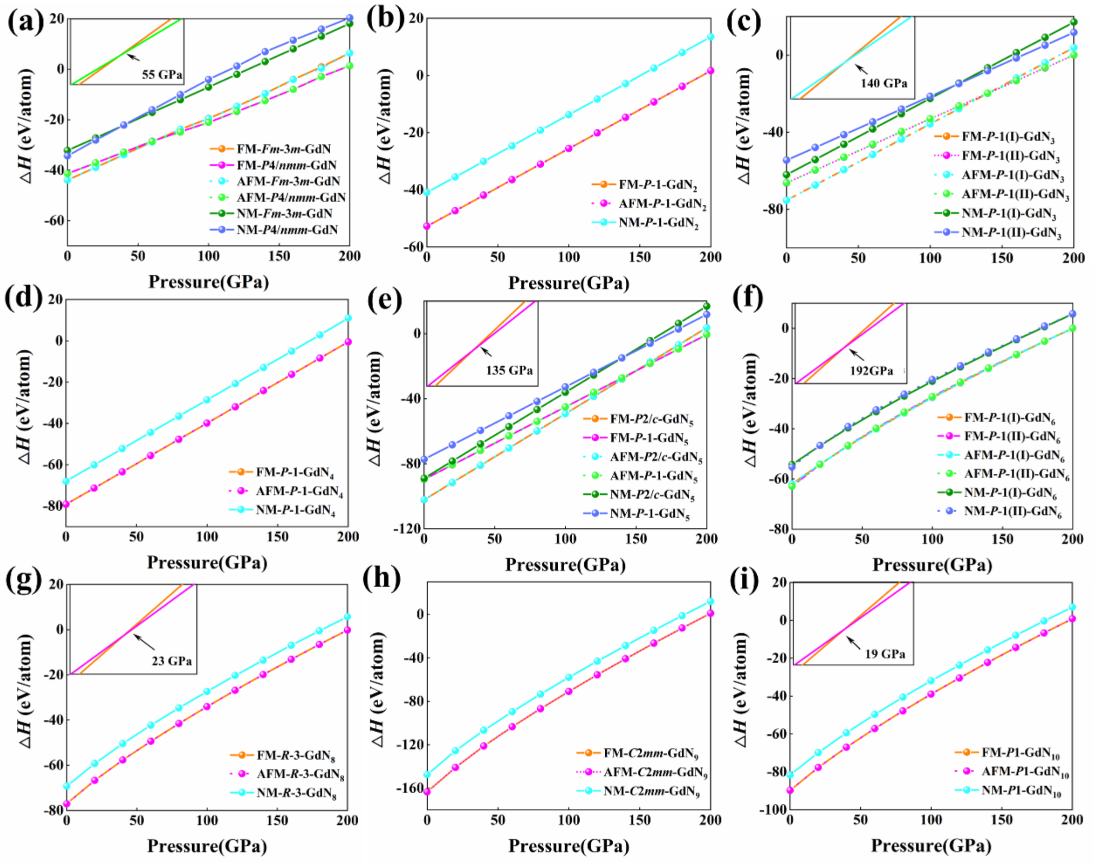

Figure S8. The enthalpy values of the ferromagnetic phase, antiferromagnetic and non-ferromagnetic phases of GdN (a), GdN_2 (b), GdN_3 (c), GdN_4 (d), GdN_5 (e), GdN_6 (f), GdN_8 (g), GdN_9 (h), and GdN_{10} (i) at different pressures.

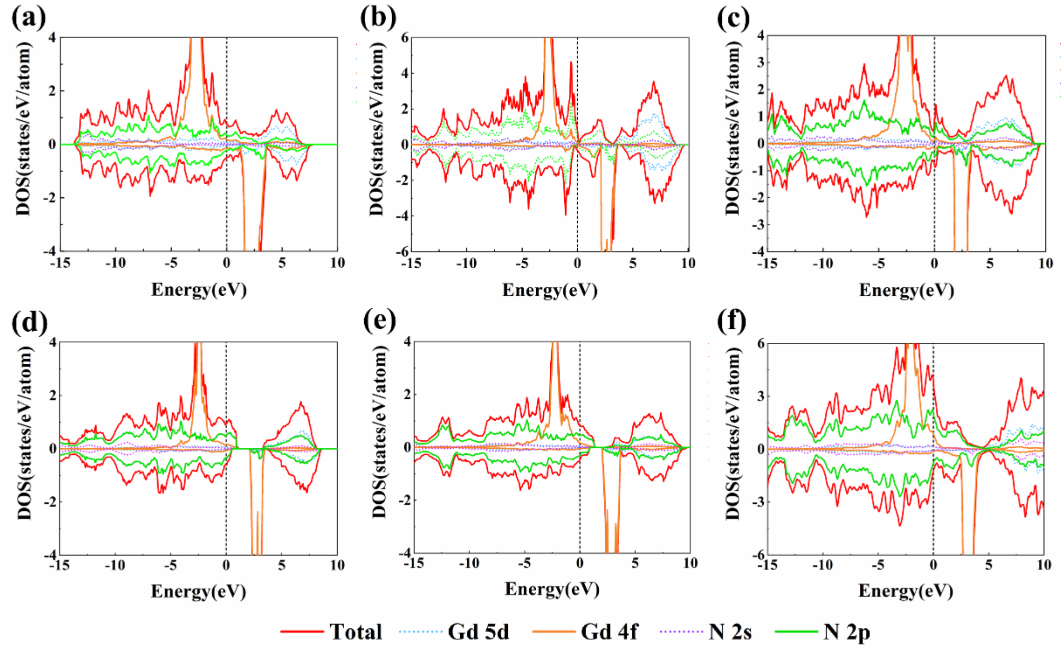

Figure S9. The total density of states (TDOS) and project density of states (PDOS) of (a) *P*-1(I)-GdN_3, (b) *P*2/*c*-GdN_5, (c) *P*-1-GdN_5, (d) *P*-1(I)-GdN_6, (e) *P*-1(II)-GdN_6, and (f) *C*2*mm*-GdN_9 at predicted pressure.

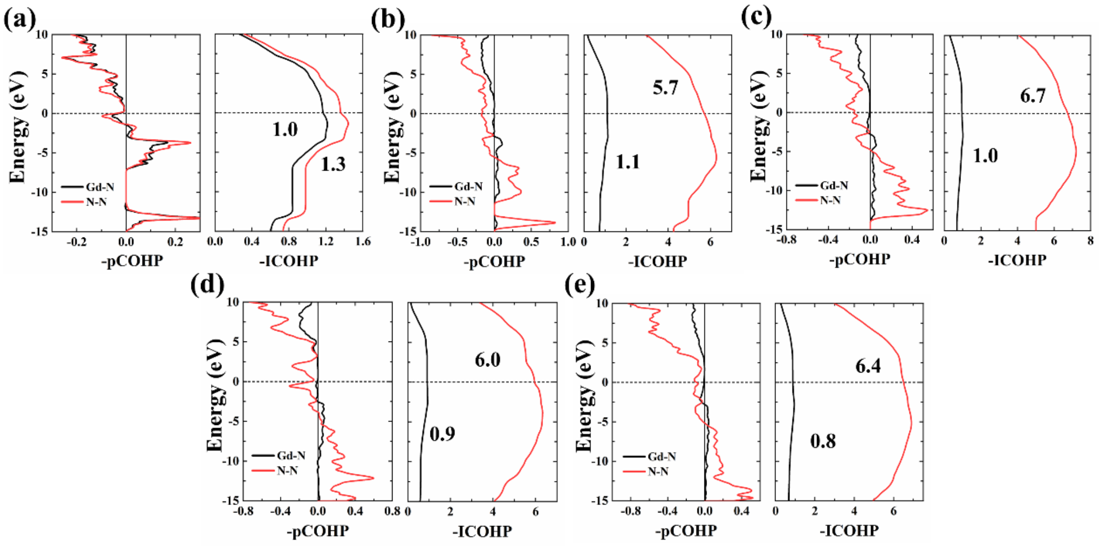

Figure S10. The -pCOHP and -ICOHP between Gd-N and N-N of *Fm*-3*m*-GdN (a), *P*-1(I)-GdN_3 (b), *P*-1(II)-GdN_3 (c), *P*2/*c*-GdN_5 (d), and *P*-1-GdN_5 (e) at predicted pressure.

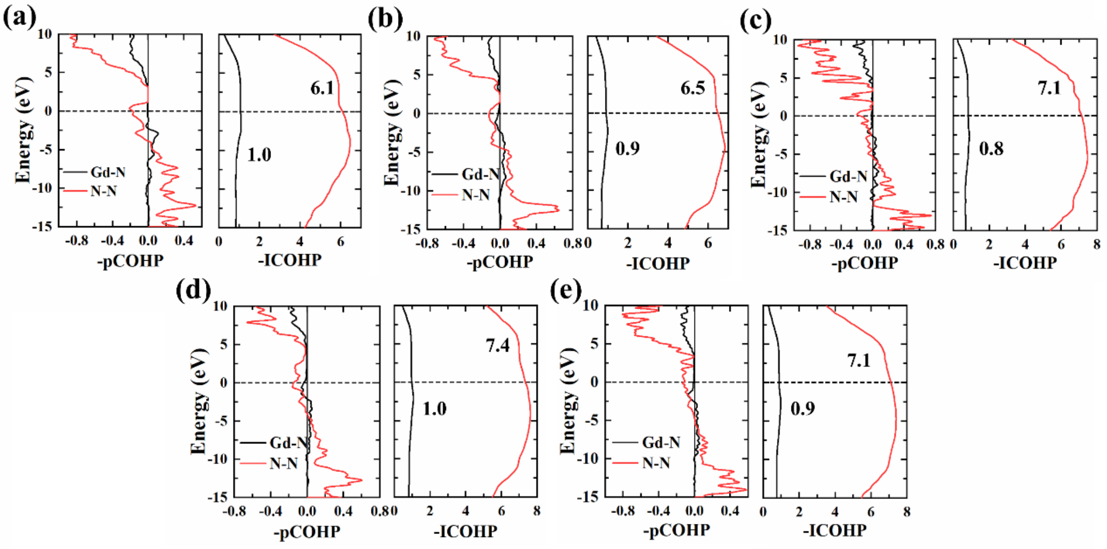

Figure S11. The -pCOHP and -ICOHP between Gd-N and N-N of *P*-1(I)-GdN_6 (a) at 100 GPa, *P*-1(II)-GdN_6 (b), *R*-3-GdN_8 (c), *C*2*mm*-GdN_9 (d), and *P*1-GdN_{10} (e) at predicted pressure.

Table S1. The N-N bond lengths of Gd-N compounds.

structures		d (Å)
P-1(I)-GdN_3	N1-N2	1.31
	N2-N3	1.32
	N1-N3	1.30
P-1(II)-GdN_3	N1-N2	1.31
	N2-N3	1.30
	N1-N3	1.28
$P2/c$-GdN_5	N1-N2	1.30
	N1-N1	1.28
	N2-N3	1.30
P-1-GdN_5	N1-N2	1.25
	N2-N3	1.34
	N3-N5	1.32
	N3-N4	1.38
	N4-N5	1.29
P-1(I)-GdN_6	N1-N1	1.33
	N1-N2	1.36
	N2-N3	1.32
P-1(II)-GdN_6	N1-N2	1.30
	N1-N3	1.35
	N1-N1	1.43
R-3-GdN_8	N1-N1	1.31
	N1-N2	1.28
$C2mm$-GdN_9	N1-N2	1.28
	N1-N3	1.28
	N2-N4	1.30
$P1$-GdN_{10}	N1-N2	1.28
	N1-N5	1.28
	N1-N10	1.28
	N3-N5	1.27
	N3-N7	1.29
	N4-N6	1.33
	N4-N8	1.31
	N6-N9	1.30
	N9-N10	1.29

Table S2. Structural parameters of predicted stable GdN_x (n=1, 3, 5) compounds.

Phase	Lattice parameters	Atomic positions
FM-*P*4/*nmm*-GdN (100 GPa)	a=b=3.829, c=2.748 Å<br>α=β=γ=90°	Gd1 (2c) (0.250, 0.250, 0.879)<br>N1 (2b) (0.750, 0.250, 0.500)
FM-*P*-1(I)-GdN_3 (100 GPa)	a=3.196, b=4.505, c=4.845 Å<br>α=99.6°, β=107°, γ=101°	Gd1 (2i) (0.080, 0.188, 0.806)<br>N1 (2i) (0.232, 0.691, 0.858)<br>N2 (2i) (0.393, 0.814, 0.671)<br>N3 (2i) (0.500, 0.636, 0.470)
AFM-*P*-1(II)-GdN_3 (200 GPa)	a=3.251, b=3.805, c=4.506 Å<br>α=102°, β=77.8°, γ=96.2°	Gd1 (2i) (0.277, 0.346, 0.252)<br>N1 (2i) (0.309, 0.952, 0.565)<br>N2 (2i) (0.171, 0.797, 0.065)<br>N3 (2i) (0.186, 0.250, 0.757)
FM-*P*2/*c*-GdN_5 (100 GPa)	a=6.560, b=3.000, c=7.035 Å<br>α=γ=90°, β=142°	Gd1 (2e) (0.000, 0.911, 0.250)<br>N1 (4g) (0.643, 0.433, 0.133)<br>N2 (4g) (0.255, 0.651, 0.143)<br>N3 (2f) (0.500, 0.915, 0.250)
FM-*P*-1-GdN_5 (200 GPa)	a=2.998, b=4.139, c=5.919 Å<br>α=102°, β=93.3°, γ=83.8°	Gd1 (2i) (0.844, 0.268, 0.208)<br>N1 (2i) (0.069, 0.122, 0.584)<br>N2 (2i) (0.294, 0.296, 0.954)<br>N3 (2i) (0.385, 0.868, 0.160)<br>N4 (2i) (0.705, 0.297, 0.677)<br>N5 (2i) (0.646, 0.707, 0.483)

Table S3. Structural parameters of predicted metastable GdN_x (n=6, 8, 9, 10) compounds.

Phase	Lattice parameters	Atomic positions
P-1(I)-GdN_6 (100 GPa)	a=3.310, b=3.692, c=4.164 Å α=74.4°, β=89.6°, γ=78.3°	Gd1 (1a) (0.000, 0.000, 0.000)<br>N1 (2i) (0.609, 0.547, 0.111)<br>N2 (2i) (0.628, 0.528, 0.619)<br>N3 (2i) (0.694, 0.882, 0.528)
P-1(II)-GdN_6 (100 GPa)	a=2.770, b=3.357, c=4.441 Å α=84.5°, β= 92°, γ=99.6°	Gd1 (1a) (0.000, 0.000, 0.000)<br>N1 (2i) (0.847, 0.313, 0.553)<br>N2 (2i) (0.616, 0.441, 0.781)<br>N3 (2i) (0.575, 0.242, 0.313)
R-3-GdN_8 (200 GPa)	a=b=c=3.699 Å α=β=γ=92.1°	Gd1 (1a) (0.000, 0.000, 0.000)<br>N1 (6f) (0.336, 0.520, 0.061)<br>N2 (2c) (0.636, 0.636, 0.636)
*C*2*mm*-GdN_9 (200 GPa)	a=4.385, b=5.739, c=4.267 Å α=β=γ=90°	Gd1 (2b) (0.500, 0.000, 0.968)<br>N1 (8f) (0.746, 0.188, 0.339)<br>N2 (4d) (0.000, 0.301, 0.339)<br>N3 (4c) (0.263, 0.000, 0.502)<br>N4 (2a) (0.000, 0.000, 0.989)
*P*1-GdN_{10} (200 GPa)	a=3.262, b=3.898, c=4.946 Å α=100°, β=93°, γ=106°	Gd1 (1a) (0.226, 0.918, 0.920)<br>N1 (1a) (0.686, 0.807, 0.179)<br>N2 (1a) (0.649, 0.475, 0.032)<br>N3 (1a) (0.089, 0.395, 0.322)<br>N4 (1a) (0.798, 0.814, 0.433)<br>N5 (1a) (0.008, 0.386, 0.062)<br>N6 (1a) (0.810, 0.084, 0.625)<br>N7 (1a) (0.390, 0.243, 0.348)<br>N8 (1a) (0.102, 0.683, 0.494)<br>N9 (1a) (0.504, 0.234, 0.599)<br>N10 (1a) (0.590, 0.541, 0.779)
